# Introduction to glycopathology: the concept, the tools and the perspectives

**DOI:** 10.1186/1746-1596-9-4

**Published:** 2014-01-20

**Authors:** Hans-Joachim Gabius, Klaus Kayser

**Affiliations:** 1 Chair of Physiological Chemistry, Faculty of Veterinary Medicine, Ludwig-Maximilians-University Munich, Veterinärstr 13, D-80539, Munich, Germany; 2 UICC-TPCC, Institut für Pathologie, Charite, Charite Platz 1, 10118, Berlin, Germany

**Keywords:** Agglutinin, Glycobiology, Glycosylation, Lectin, Neoglycoconjugates

## Abstract

**Virtual slides:**

The virtual slides for this article can be found here: http://www.diagnosticpathology.diagnomx.eu/vs/1670639891114983.

Analyzing the flow of biological information is a fundamental challenge for basic sciences. The emerging results will then lend themselves to the development of new approaches for medical applications. Toward this end, the products of protein/lipid glycosylation deserve special attention. The covalent attachment of sugars to these carriers means much more than just a change of the carriers’ physicochemical properties. In principle, the ubiquitous presence of glycoconjugates and the close inspection of the particular structural ‘talents’ of carbohydrates provide suggestive evidence for information coding by sugars. In fact, the theoretical number of ‘words’ (oligomers) formed by ‘letters’ (monosaccharides) is by far higher than by using nucleotides or amino acids. In other words, glycans harbor an unsurpassed coding capacity. The cyto- and histochemical detection of dynamic changes in the profile of cellular glycans (glycome, the equivalent of the proteome) by sugar receptors such as antibodies used as tools underscores the suitability of carbohydrates for such a task. The resulting staining patterns can be likened to a molecular fingerprint. By acting as ligand (counterreceptor) for endogenous receptors (tissue lectins), glycan epitopes become partners in a specific recognition pair, and the sugar-encoded information can then be translated into effects, e.g. in growth regulation. Of note, expression of both sides of such a pair, i.e. lectin and cognate glycan, can physiologically be orchestrated for optimal efficiency. Indeed, examples how to prevent autoimmune diseases by regulatory T cells and restrict carcinoma growth by a tumor suppressor attest occurrence of co-regulation. In consequence, these glycans have potential to establish a new class of functional biomarkers, and mapping presence of their receptors is warranted. In this review, the cyto- and histochemical methods, which contribute to explore information storage and transfer within the sugar code, are described. This introduction to the toolbox is flanked by illustrating the application of each type of tool in histopathology, with focus on adhesion/growth-regulating galectins. Together with an introduction to fundamental principles of the sugar code, the review is designed to guide into this field and to inspire respective research efforts.

## Introduction

**Figure 1:**
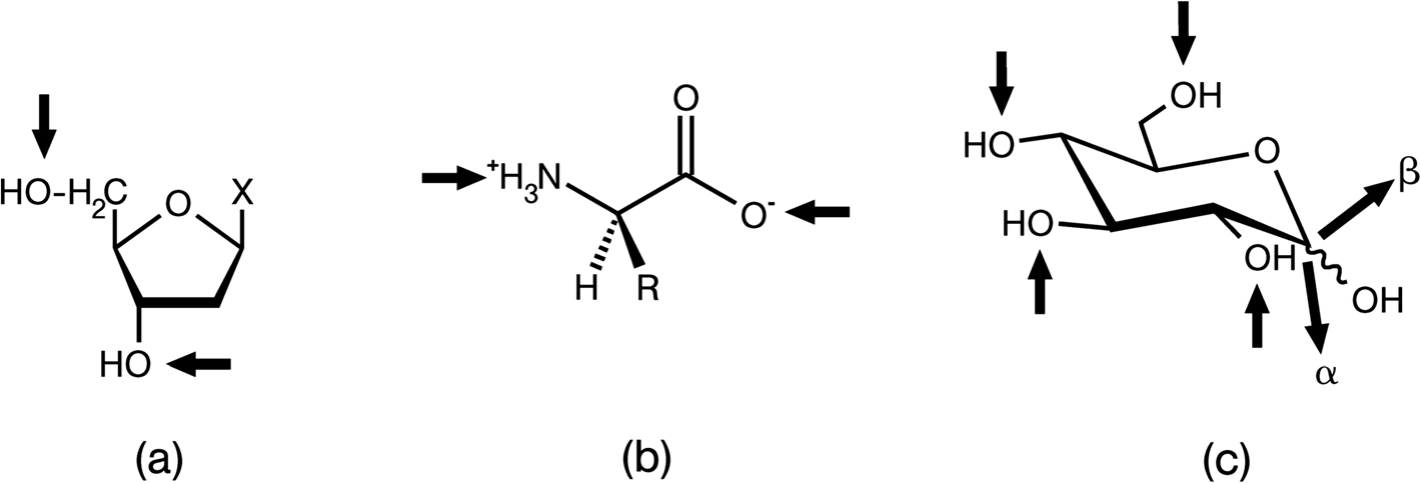
**Illustration of the linkage points for oligomer formation in biomolecules by arrows.** The phosphodiester bond in nucleic acid biosynthesis **(a)** and the peptide bond in protein biosynthesis **(b)** yield linear oligomers. In contrast, the glycosidic linkage in oligosaccharides can involve any hydroxyl group, opening the way to linear and also branched structures **(c)**. Using d-glucose (Glc) as an example, its active form UDP-Glc allows conjugation of this sugar to carbohydrate acceptors to any hydroxyl group, as symbolized by arrows directed towards the hydroxy groups (for list of resulting diglucosides, please see Table 1). The anomeric position in chain elongation can vary, as symbolized by two bold arrows pointing away from the molecule (from [1], with permission).

Basic biochemistry teaches that the flow of biological information starts with transcription of genes, which code for proteins or RNA-based regulators of protein production. Both nucleotides and amino acids are linked to oligo- and polymers by the assembly-line production of linear chains. As shown in Figure 1a, b, this means that the only parameter varied in a respective biopolymer is the sequence of the building blocks. In consequence, the range of sequence permutations defines the coding capacity of these biomolecules. For a trimer, the natural set of four nucleotides will result in 64 (4^3^) isomers, the alphabet of 20 amino acids in 8000 (20^3^) tripeptides. For coding biological information in a minimum of space (such constraints become a major issue on cell surfaces, due to the requirement to present a wide array of signals), the coding capacity (number of distinct ‘words’ generated by the ‘letters’ of the respective alphabet) needs to reach far higher values. In molecular terms, one way toward this aim is to generate structural diversity beyond the sequence. How can this be accomplished by comparatively easy biochemistry, as realized in the cases of nucleic acids and proteins? Which type of biomolecule can meet this demand and also has the suited wide distribution in Nature? These questions will be answered in the next section by turning to biomolecules well-known for their capacity to store energy, i.e. the carbohydrates.

### Carbohydrates: the third alphabet of life

**Figure 2:**
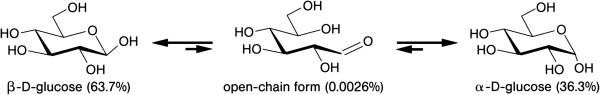
**Illustration of the equilibrium including the two anomeric forms of d-glucose.** The percentages of presence of the two anomeric hexopyranose and the open-chain forms in equilibrium are given in the bottom line (from [1], with permission).

A brief inspection of the structure of the most common sugar is enormously illuminating: *D*-Glucose (Glc) introduces the reader to i) the presentation of chemically rather equal hydroxyl groups for versatile formation of glycosidic linkages and ii) the principle of anomery, a type of isomery concerning the constellation at the anomeric center (the C1 atom) after ring formation (for details, please see Figure 2). As consequence, each hydroxyl group can be engaged as acceptor. Activation of a sugar at the anomeric center (here UDP-Glc) generates the donor, present in two anomeric constellations (α or β). The site-specific coupling reaction is driven by a glycosyltransferase (for an illustration with *D*-Glc as example, please see Figure 1c) [1]. A resulting diglucoside therefore is not simply characterized as Glc-Glc as is the case for a dipeptide. Information on the anomeric character (α or β) and the two connection points (theoretically possible are 1,1; 1,2; 1,3; 1,4; 1,6) is necessary for describing the individual disaccharide (to give an example, maltose is Glcα1,4Glc; for a listing of all naturally occurring diglucosides, please see Table 1).

**Table 1:** Naturally occurring disaccharides formed from two glucose units

**Type of linkage**	**Common name**
α1 → 2	kojibiose
β1 → 2	sophorose
α1 → 3	nigerose
β1 → 3	laminaribiose
α1 → 4	maltose
β1 → 4	cellobiose
α1 → 6	isomaltose
β1 → 6	gentiobiose
α1↔α1	trehalose

All disaccharides are conversion or degradation products of natural polysaccharides and glycosides, except for trehalose which is present in bacteria, fungi and insects; arrows depict involvement of anomeric centers (from [1], with permission).

In addition, as further sources of variability, the ring size (furanose or pyranose) can differ, branching of chains is possible and, like in proteins, substitutions (e.g. phosphorylation) can be introduced. Assuming an alphabet size of 20 for model calculations, the sugars (the third alphabet of life) can theoretically build 11.264×10^6^ trimers, compared to 64/8000 for the first (nucleotides) or second (amino acids) alphabets [2]. Even though the donor-acceptor biochemistry restricts the panel size, the comparison given in Figure 1 graphically documents the second-to-none capacity of carbohydrates for coding. Nonetheless, compared to the set of nucleotides and amino acids, less is commonly taught in biochemistry courses about the elements of the sugar alphabet.

**Figure 3:**
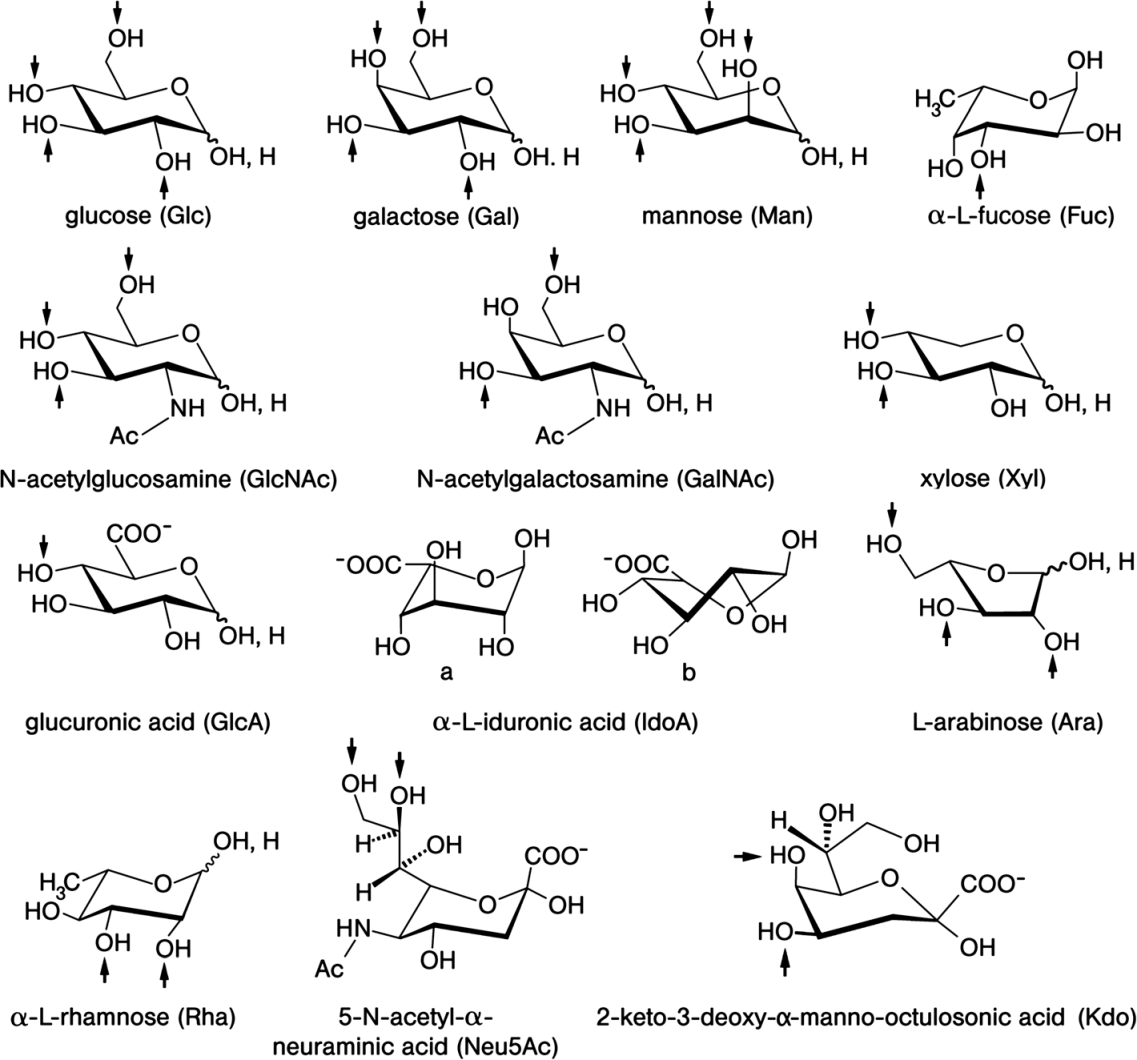
**Illustration of the alphabet of the sugar language.** Structural representation, name and symbol as well as the set of known acceptor positions (arrows) in glycoconjugates are given for each letter. Four sugars have l-configuration: fucose (6-deoxy-l-galactose), rhamnose (6-deoxy-l-mannose) and arabinose are introduced during chain elongation, whereas l-iduronic acid (IdoA) results from post-synthetic epimerization of glucuronic acid at C-5. The ^1^C_4_ conformation of IdoA **(a)** is in equilibrium with the ^2^S_O_ form **(b)** in glycosaminoglycan chains where this uronic acid can be 2-sulfated (please see Figure 7d). All other “letters” are d-sugars. Neu5Ac, one of the more than 50 sialic acids, often terminates sugar chains in animal glycoconjugates. Kdo is a constituent of lipopolysaccharides in the cell walls of Gram-negative bacteria and is also found in cell wall polysaccharides of green algae and higher plants. Foreign to mammalian glycobiochemistry, microbial polysaccharides contain the furanose ring form of d-galactose and also d/l-arabinose indicated by an italic “*f*” derived from the heterocyclus furan. The α-anomer is prevalent for the pentose arabinose, e. g. in mycobacterial cell wall arabinogalactan and lipoarabinomannan. β1,5/6-Linked galactofuranoside is present in the arabinogalactan and the β1,3/6 linkage in lipopolysaccharides (from [1], with permission).

**Figure 4:**
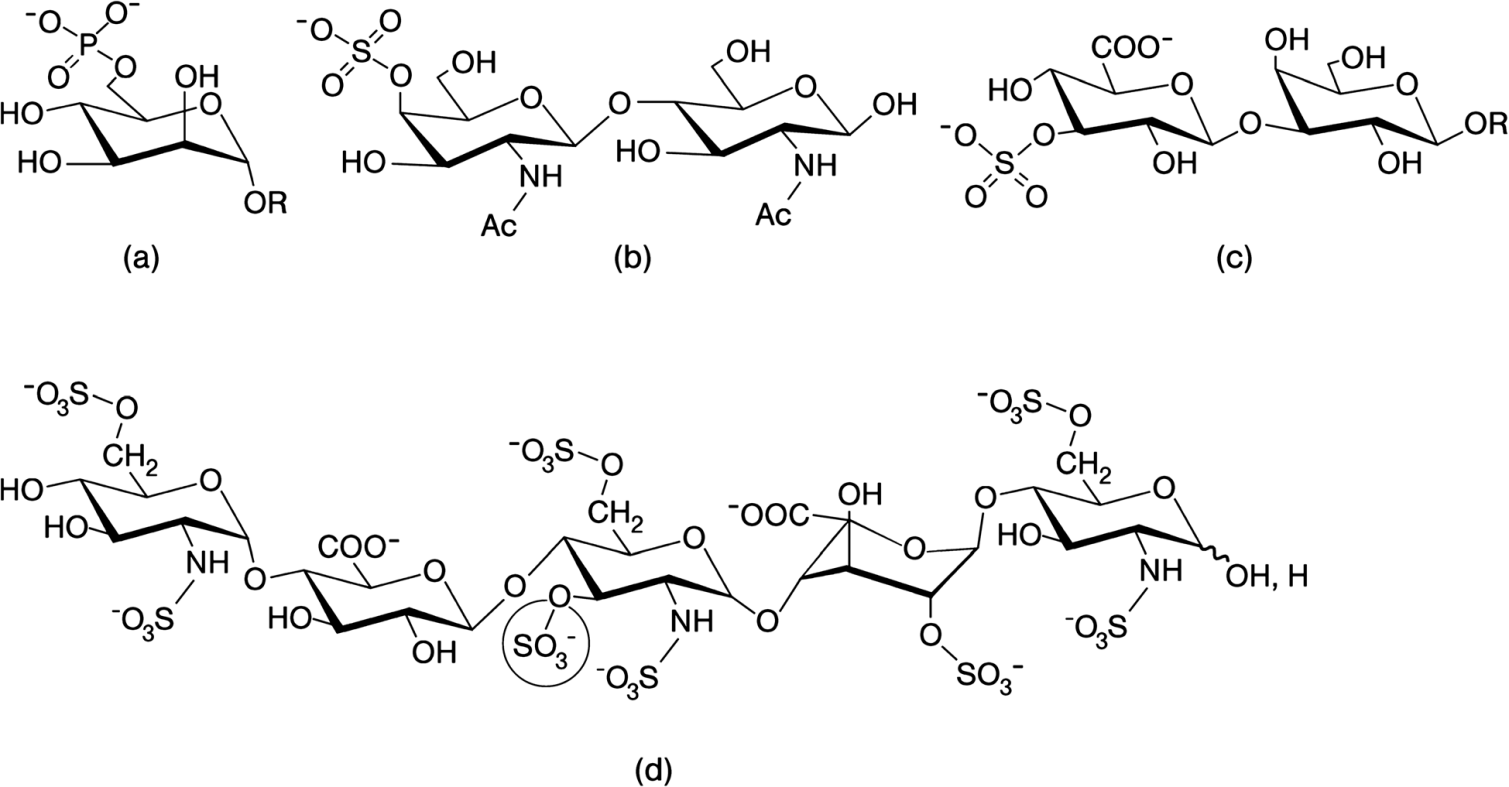
**Illustration of phosphorylated (phosphated) and sulfated (sulfurylated) glycan “words”.** 6-Phosphorylation of a mannose moiety (in the context of a mannose-rich pentasaccharide) is the key section of a routing signal in lysosomal enzymes **(a)**, 4-sulfation of the GalNAcβ1,4GlcNAc (LacdiNAc) epitope forms the “postal code” for clearance from circulation by hepatic endothelial cells of pituitary glycoprotein hormones labeled in such a way **(b)**, the HNK-1 (human natural killer-1) epitope (3-sulfated GlcAβ1,3Galβ1,4GlcNAc) is involved in cell adhesion/migration in the nervous system **(c)** and the encircled 3-O-sulfation in the pentasaccharide’s center is essential for heparin’s anti-coagulant activity **(d)**. All sugars are in their pyranose form. Please note that the central glucosamine unit has N,O-trisulfation and that the 2-sulfated IdoA, given in the ^1^C_4_ conformation, can also adopt the hinge-like ^2^S_O_ skew-boat structure (please see Figure 3; about 60% or more for the ^2^S_O_ form in equilibrium depending on the structural context) when present within glycosaminoglycan chains of the proteoglycan heparin. 2-Sulfation of IdoA serves two purposes: favoring the hinge-like ^2^S_O_ conformation and precluding re-conversion to GlcA (from [1], with permission).

In Nature, the sugar alphabet starts with Glc (building block of starch and cellulose, which differ in anomeric linkage type of the 1,4-connected units, highlighting the importance of seemingly small structural changes) and its 2’-derivative N-acetyl-glucosamine (GlcNAc, unit of chitin) (Figure 3). These biomacromolecules actually are the most abundant polymers on earth (please see below). Epimers of Glc (a switch of a hydroxyl group from the energetically preferred equatorial to the axial position; please see the 4’-epimer galactose (Gal) and the 2’-epimer mannose (Man) in Figure 3), deoxy sugars, uronic acids and other anionic carbohydrates further belong to the sugar alphabet (Figure 3). Linking the ‘letters’ to ‘words’ will proceed without a genetic blueprint. Instead, factors such as substrate availability play into the product panel. The overall coding capacity in oligosaccharides is further enhanced, as for proteins, by introducing substitutions such as phosphorylation and sulfation (for physiologically prominent examples, please see Figure 4). In aggregate, the sugar alphabet, by virtue of the distinct chemical properties of sugars, enables enormous structural variability beyond the sequence: anomeric status, linkage formation involving different acceptor sites and ring size, with frequent occurrence of branching and site-specific presence of modifications, make this possible. Thus, the coding capacity of the sugar language is orders of magnitude higher than for nucleic acids and proteins.

Carbohydrate oligomers (glycans) or polymers (polysaccharides) are ubiquitous. Cellulose and chitin are the two most abundant organic compounds (with estimations for chitin production by animals reaching at least 10 gigatons) [3]. On eukaryotic cell surfaces, the glycan part of glycoconjugates (hybrid molecules of sugar and lipid/protein, termed glycoproteins/proteoglycan or glycolipid [4-8] is prominently positioned and readily accessible. The formation of at least 41 bonds between sugar and protein, with eight amino acids and 13 sugars involved, highlights the wide diversity of these types of conjugation in Nature [9]. Necessarily, an intricate enzymatic machinery for glycan assembly and remodeling (by glycosyltransferases, glycosidases and substitution-introducing enzymes such as sulfotransferases [4-8,10]) has developed to realize the actually presented set of glycans on cells (glycome) from the panel of theoretically possible glycans. In human cells, proteins destined for cell surface residence or secretion commonly carry glycan chains at the asparagine: Asn of the glycosylation sequon (Asn-X-Ser/Thr; X≠Pro), the amide’s nitrogen (N) acting as acceptor (thus the term N-glycosylation). But this high-level complexity in structure comes at a scientific price: “in this remarkable age of genomics, proteomics, and functional proteomics, I am often asked by my colleagues why glycobiology has apparently lagged so far behind the other fields. The simple answer is that glycoconjugates are much more complex, variegated, and difficult to study than proteins or nucleic acids” [11]. To address this problem and set the stage for full-scale glycome analysis led to development of sophisticated analytical procedures, even separating and identifying structural isomers reliably [12]. Their availability opens the door to comparative studies of glycan sequences in clinical specimens in the quest to define glycobiomarkers for diseases such as cancer, CA19-9 being an example [13-15].

In cyto- and histochemistry, the methods to detect glycan presence gained in specificity over time. Initially, carbohydrates were visualized by applying protocols like the PAS procedure (based on oxidizing vicinal hydroxyl groups of carbohydrates to reactive aldehydes) or stains such as Alcian blue (for glycosaminoglycans). These methods were replaced with marked increase in specificity by applying sugar receptors [16-21]. Known from transfusion medicine, certain antibodies are reactive with carbohydrate epitopes such as the blood group ABH determinants [22], and the monoclonal-antibody technology accounts for access to custom-made tools. The second class of sugar receptors used as laboratory tools for glycan detection are non-immunoglobulin agglutinins. They are referred to this way due to their capability to aggregate erythrocytes (haemagglutination) in a sugar-dependent manner [23,24]. These receptor proteins, separated from the class of antibodies and from enzymes, which process the bound sugar, are called lectins (for an overview on the history of lectinology starting with detection in snake venom and plant extracts, please see [25]). The large number of lectins from plants and invertebrates makes detailed monitoring of distinct glycome aspects possible, e.g. on status and type of sialylation or substitution of the core of N-glycans [16-21]. Especially these features are susceptible to changes upon altering genetic or microenvironmental parameters [26-28]. Sialylation can physiologically mask a glycan chain, the addition of the bisecting GlcNAc residue (a molecular ‘wedge’ between two glycan branches) or a fucose moiety to the N-glycan core is not a merely subtle structural difference. Instead, it is a molecular switch for the glycan’s conformational dynamics, with important functional consequences during and after glycoprotein maturation [29-34]. What this popular method of specific detection of glycan epitopes also teaches is that these structures can serve as ligands (counterreceptors) in molecular recognition. Glycan detection by lectins (as tools) thus prompts the question on the presence of endogenous receptors, to read the sugar-encoded information and translate it into cellular responses.

### Lectins: translators of the sugar code

The wide diversity of glycan structures in Nature let us expect an equally broad set of lectins. The diversity should concern both the number of families, defined by the folding pattern, and the number of members in each family. On the level of protein folds with capacity to bind sugars, this expectation is borne out: at least 14 types have developed in phylogenesis to endow animal/human proteins with lectin activity (Table 2). Once established, each domain has undergone evolutionary diversification to generate a lectin family. This holds true for all cases listed in Table 2. Tracing the routes to diversity in individual families, e.g. C-type lectins and galectins, gives an impression on the ways sequences in the carbohydrate recognition domains (CRD) and their spatial modes of presentation can diverge including occurrence of single nucleotide polymorphisms in lectin genes [35-39]. Formation of non-covalent aggregates or the tandem-repeat display underlie the lectins’ cross-linking capacity, depending on the presence of matching glycans. In this respect, the binding of lectins to their cellular targets markedly benefits from a special property of glycans: acquisition of only few energetically preferred conformers by limited flexibility [40,41]. In contrast, peptides will adopt certain conformations only when embedded into the context of a protein, otherwise be highly flexible. In other words, glycans often acquire key-like conformations. They are suited to be accommodated into a complementary binding site (CRD; molecular lock). This favorable factor for the thermodynamics of binding adds to the advantageous features of glycans in information coding outlined above.

**Table 2:** Overview of folds with capacity to bind sugars and of lectin classes

**Type of fold**	**Example for lectin**	**Example for ligand**
β-sandwich (jelly-roll)	a) galectins	β-galactosides
	b) calnexin, calreticulin	Glc_1_Man_9_Glc*N*Ac_2_
	c) ERGIC-53, VIP36, VIPL	Man_x_Glc*N*Ac_2_
	d) CRD^a^ of Fbs1 in SCF E3 ubiquitin ligase and peptide-*N*-glycanase	Man_3_Glc*N*Ac_2_; mannopentaose
	e) pentraxins	glycosaminoglycans, MOβDG, 3-sulfated Gal, Gal*N*Ac and GlcA, Man-6-phosphate
	f) G-domains of the LNS family (laminin, agrin)	heparin
C-type	asialoglycoprotein receptor, collectins, selectins	Fuc, Gal, Gal*N*Ac, Man, heparin tetrasaccharide
I-type (Ig fold)	N-CAM, TIM-3, siglecs	Man_6_Glc*N*Ac_2_, HNK-1 epitope, α2,3/6-sialylated glycans
P-type	mannose-6-phosphate receptors (MR) and proteins with MR homology domain (erlectin, OS-9)	Man-6-phosphate, Man_5,8_Glc*N*Ac_2_
β-trefoil	a) fibroblast growth factors	heparan sulfate
	b) cysteine-rich domain of C-type macrophage mannose receptor	Gal*N*Ac-4-sulfate in Lacdi*N*Ac
	c) lectin domain in Gal*N*Ac-Ts^b^ involved in mucin-type O-glycosylation	Gal*N*Ac
	d) hemolytic lectin CEL-III of sea cucumber and lectin EW29 of earthworm	Gal
β-propeller	a) 4-bladed: tachylectin-3	S-type lipopolysaccharide Glc*N*Ac/Gal*N*Ac
	b) 5-bladed: tachylectin-2	
	c) 6-bladed: tachylectin-1	KDO
β-prism I	secretory proteins zg16p/b	Man, heparan sulfate
β-prism II	pufferfish (fugu) lectin	Man
β-barrel with jelly-roll topology	tachylectin-4, eel (*Anguilla anguilla*) agglutinin, X-epilectin	Fuc
Fibrinogen-like domain	a) ficolins	Glc*N*Ac
	b) intelectins (mammalian, *Xenopus*)	Gal*f*, pentoses
	c) tachylectin-5	*N*-acetylated sugars sialic acid
	d) slug (*Limax flavus*) lectin	
Link module	CD44, TSG-6, LYVE-1, aggregating proteoglycans	hyaluronic acid
Hevein-like domain	Tachycytin and spider (*Selenocosmia huwena*) neurotoxin; cobra venom cardiotoxin	Gal*N*Ac; heparin-derived disaccharide
(β/α)_8_ barrel (glycoside hydrolase family 18)	YKL-40 (human cartilage glycoprotein-39; chitinase-like lectin)	(Glc*N*Ac)_n_
Short consensus repeat (complement control protein module)	Factor H (complement regulator)	glycosaminoglycans, sialic acid

^a^carbohydrate recognition domain, ^b^*N*-acetylgalactosaminyltransferases; adapted from [42], with permission.

Turning back to the protein side, genes for CRDs are widely disseminated in the genome. As integral part of many proteins, these domains can even be linked to other functional sites. This design leads to multifunctional proteins, e.g. collagen-like tails for aggregation in innate immunity lectins or cell- and carbohydrate-binding sites in a slime mold lectin working together to effect ordered cell migration [43-45]. Evidently, homing in on distinct targets on bacterial surfaces or executing a certain task in guiding cells can take more than being able to bind a certain sugar. Intriguingly, lectin reactivity of glycoconjugates in their physiological context can be markedly modulated. In total, structural and topological factors of glycans on six levels cooperate to let lectins find their natural counterreceptors [41]. The attained specificity in target recognition lays the foundation for the spectrum of lectin functions compiled in Table 3. Exemplarily looking at routing/delivery processes, cargo selection and transport via lectins, e.g. of enzymes with the mannose-6-phosphate signal (Figure 4a) to lysosomes, glycoproteins with multiantennary N-glycans to the apical side of polarized cells and of a cell adhesion molecule (L1) to axonal membranes [46-49], illustrate the reason why glycans are likened to a postal code. Orchestration of glycan/lectin expression goes far beyond transport processes.

**Table 3:** **Functions of animal and human lectins**^**a**^

**Activity**	**Example of lectin**
Recognition of stem region of *N*-glycans, a signal for ubiquitin conjugation when accessible in incorrectly folded glycoproteins	F-box proteins Fbs1 and Fbs2, which comprise the ligand-specific part of SCF^b^ ubiquitin ligase complexes
Molecular chaperones with dual specificity for Glc_2_/Glc_1_Man_9_Glc*N*Ac_2_ and protein part of nascent glycoproteins in the ER	Malectin/ribophorin I complex, calnexin, calreticulin
Targeting of misfolded glycoproteins with Man_8-5_Glc*N*Ac_2_ as carbohydrate ligand to ER-associated degradation (ERAD)	EDEM1,2^c^/Mnl1 (Htm1) (lectins or glycosidases?), Yos9p (MRH^d^ domain) in yeast, erlectin (XTP3-B^e^) and OS-9^f^ in mammals
Intracellular routing of glycoproteins and vesicles and apical delivery	Comitin, ERGIC53^g^ and VIP36^h^ (probably also ERGL^i^ and VIPL^j^), galectins-3, -4 and -9, P-type lectins
Intracellular transport and extracellular assembly	Non-integrin 67 kDa elastin/laminin-binding protein
Enamel formation and biomineralization	Amelogenin
Inducer of membrane superimposition and zippering (formation of Birbeck granules)	Langerin (CD207)
Cell type-specific endocytosis	Cysteine-rich domain (β-trefoil) of the dimeric form of mannose receptor for Gal*N*Ac-4-SO_4_-bearing glycoprotein hormones in hepatic endothelial cells, dendritic cell and macrophage C-type lectins (mannose receptor family members (tandem-repeat type) and single-CRD^k^ lectins such as trimeric langerin/CD207 or tetrameric DC-SIGN^l^/CD209), hepatic and macrophage asialoglycoprotein receptors, HARE^m^, P-type lectins
Recognition of foreign glycans (β1,3-glucans, cell wall peptidoglycan, LOS^n^ and LPS^o^), mycobacterial glycolipid or host-like epitopes	CR3^p^ (CD11b/CD18, Mac-1 antigen), C-type lectins such as collectins, DC-SIGN, dectin-1, Mincle and RegIIIγ (murine)^q^ or HIP/PAP (human), ficolins, galectins, immulectins, intelectins, *Limulus* coagulation factors C and G, siglecs, tachylectins
Recognition of foreign or aberrant glycosignatures on cells (including endocytosis or initiation of opsonization or complement activation) and of apoptotic/necrotic cells (glycans or peptide motifs)	Collectins, C-type macrophage and dendritic cell lectins, CR3 (CD11b/CD18, Mac-1 antigen), α/Θ-defensins, ficolins, galectins, pentraxins (CRP, limulin), RegIIIγ (HIP/PAP), siglecs, tachylectins
Targeting of enzymatic activity in multimodular proteins	Acrosin, *Limulus* coagulation factor C, laforin, β-trefoil fold ((QxW)_3_ domain) of Gal*N*Ac-Ts^r^ involved in mucin-type *O*-glycosylation, frequent in microbial glycosylhydrolases for plant cell wall polysaccharides, termed carbohydrate-binding modules
Bridging of molecules	Cerebellar soluble lectin, cytokines (e.g. IL-2^s^–IL-2R and CD3 of TCR), galectins
Induction or suppression of effector release (H_2_O_2_, cytokines etc.)	Chitinase-like YKL-40, galectins, I-type lectins (e.g. CD33 (siglec-3), siglecs-7 and -9), selectins and other C-type lectins such as CD23, BDCA2 and dectin-1, Toll-like receptor 4
Alteration of enzymatic activities in modular proteins/receptor endocytosis via lattice formation	Mannan-binding lectin (acting on meprins); galectins
Cell growth control, induction of apoptosis/anoikis and axonal regeneration	Amphoterin and other heparin-binding proteins, cerebellar soluble lectin, chitinase-like lectins, C-type lectins, galectins, hyaluronic acid-binding proteins, siglecs (e.g. CD22 and CD33)
Cell migration and routing	Galectins, hyaluronic acid-binding proteins (CD44, hyalectans/lecticans, RHAMM^t^), I-type lectins, selectins and other C-type lectins
Cell–cell interactions	Galectins, gliolectin, I-type lectins (e.g. siglecs, N-CAM^u^, P_0_ or L1), selectins and other C-type lectins such as DC-SIGN or macrophage mannose receptor
Cell–matrix interactions	Calreticulin, discoidin I, galectins, heparin- and hyaluronic acid-binding lectins including hyalectans/lecticans
Matrix network assembly	Galectins (e.g. galectin-3/hensin), non-integrin 67 kDa elastin/laminin-binding protein, proteoglycan core proteins (C-type CRD and G1 domain of hyalectans/lecticans)

^a^adapted from [42], with permission, and extended.

^b^Skp-1-Cul1-F-box protein complex.

^c^ER degradation enhancing α-mannosidase-like protein.

^d^mannose-6-phosphate receptor homology.

^e^XTP3-transactivated gene B precursor.

^f^osteosarcoma 9.

^g^ER-Golgi intermediate compartment protein (lectin) (MW: 53 kDa).

^h^vesicular-integral (membrane) protein (lectin) (MW: 36 kDa).

^i^ERGIC-53-like protein.

^j^VIP-36-like protein.

^k^carbohydrate recognition domain.

^l^dendritic cell-specific ICAM-3-grabbing nonintegrin.

^m^hyaluronan receptor for endocytosis.

^n^lipooligosaccharide.

^o^lipopolysaccharide.

^p^complement receptor type 3.

^q^member of regenerating (reg) gene family of secreted proteins.

^r^UDP-GalNAc:polypeptide *N*-acetylgalactosaminyltransferases.

^s^interleukin-2.

^t^receptor for hyaluronan-mediated motility.

^u^neural cell adhesion molecule.

In tumor biology, a deficiency in suppressor activity can cause the release from growth control. The study of reconstitution of activity of the tumor suppressor p16^INK4a^*in vitro* in pancreas carcinoma cells (Capan-1) has revealed the co-regulation of glycogenes (encoding several glycosyltransferases and two enzymes in sialic acid biosynthesis, a sugar at terminal positions regulating recognition [50]) with two distinct lectins (transcriptionally and post-transcriptionally) in order to make cells susceptible for lectin-dependent anoikis induction [51-53]. In detail, decreased synthesis of sialic acid reduces α2,6-sialylation on the cell surface, this in turn favoring binding and cross-linking of the fibronectin receptor (α_5_β_1_-integrin) by galectin-1 and the ensuing activation of caspase-8 [51,53]. Expression of the physiological antagonist (galectin-3) is decreased for optimal cellular responsiveness to galectin-1. This homodimeric galectin is also the trigger element for growth regulation in other cases, with a different counterreceptor. Instead of a glycoprotein, binding of ganglioside GM1 in neuroblastoma cells and activated effector T cells, in both cases after enzymatic conversion of the precursor GD1a into the active counterreceptor by a sialidase [54-58], starts the regulatory cascade. The second sialic acid moiety of ganglioside GD1a (please see Figure 3 for structure of sialic acid) at its hexasaccharide terminus thus impairs the reactivity of the entire sugar chain of the ganglioside to preclude lectin recognition, its removal facilitating the binding to galectin-1.

Evidently, dynamic remodeling of glycans on the cell surface is an efficient means to let cells swiftly become responsive to distinct lectins, without the need for a complete neosynthesis. The specificity of counterreceptor recognition by tissue lectins guarantees correct reading of signals. As the *in vitro* examples indicate, lectins are expressed not only by normal cells but also in malignancy. Purification from extracts of tumor cells or tissue using affinity chromatography provides material for characterization and functional testing [59,60]. Given this expression of the glycobiological effectors in tumors, a new research area is opened, with the potential to identify a prognostic indicator and targets for therapy [61,62]. To test these assumptions, the developments of experimental approaches to detect lectin presence (alone and embedded in a network) and the expression of binding partners – in view of the mentioned cases for orchestration of both sides of this recognition system – are the central challenges. They are mastered by strategic combination of chemistry and biochemistry, adapted to cyto- and histochemistry.

### How to detect tissue lectins

Having described glycans as information-bearing biomolecules and plant/invertebrate lectins as tools to characterize the cellular glycophenotype and disease-associated alteration (please see first section in Table 4), the principle to follow for the design of lectin-detecting probes is rather obvious: use glycans in search of binding sites (CRDs). Products from chemical synthesis of oligosaccharides (for an introduction into this field, please see [63]) or from purification from natural sources such as glycoproteins can fulfill any request for product specifications. Next, the issues of turning glycans into high-affintiy binders and of introducing a label are addressed. As the natural role model for glycan presentation in clusters teaches, the conjugation of a carbohydrate derivative to a scaffold (protein or synthetic polymer) is a suitable strategy to achieve the affinity increase. This synthetic attachment leads to neoglycoproteins/neoglycoconjugates (the prefix *neo* describing the synthetic origin), with the expected enhancement of lectin reactivity when moving from mono- to polyvalency [64-67]. Having prepared the conjugate, introducing a label is the final step of the synthetic scheme. A natural mode of labeling is the association of glycan presentation with an enzymatic activity, e.g. in alkaline phosphatase or horseradish peroxidase [68-70]. This principle can be adopted by preparing enzyme-neoglycoprotein conjugates or neoglycoenzymes [71,72]. Of note, the reactivity of the high-mannose-type N-glycans of the plant peroxidase, a component of staining kits, with mannose-specific lectins *in situ*, e.g. the C-type tandem-repeat-type receptor on macrophages, gives reason to perform rigorous controls for specificity to exclude a false-positive outcome in cyto- and histochemical processing.

**Table 4:** The four classes of reagents used in glycocyto- and histochemical analysis

**Experimental aim**	**Type of reagent**	**Example**
Detect certain aspects of glycosylation	Plant lectin/carbohydrate-specific antibody	Monitoring the presence of β1,6-branching in N-glycans or of sialylated Lewis epitopes
Detect accessible sites binding a distinct carbohydrate epitope	Neoglycoconjugate	Detecting binding sites for sialylated Lewis epitopes in colon cancer
Detect distinct lectins *in situ*	Antibody specific for endogenous lectin	Performing immunohistochemical galectin fingerprinting in colon cancer (with prognostic relevance for galectins)
Detect accessible ligands (glycan/peptide) for an endogenous lectin	Tissue lectin	Delineating prognostic relevance for galectin-3 binding in head and neck cancer sections

From [73], with permission.

In general, chemistry facilitates to introduce any label such as biotin or fluorescent dyes into the scaffold to let the neoglycoconjugates enter routine practice. This approach is referred to as glycocyto- and histochemistry (please see second section in Table 4). Unless receptors are blocked by their engagement with high-affinity tissue sites or harmed by processing steps such as fixation, presence of carbohydrate-binding proteins can then be comprehensively studied. Using the same type of scaffold and label, in combination with stringent controls (testing carbohydrate-free scaffold and running inhibition assays), will enable a cell/tissue profiling of capacity to bind carbohydrate epitopes. Histopathologic application of these tools not only ascertained the validity of the concept but also indicated the potential to distinguish cell/tumor types [74-77]. At 95% sensitivity/72% specificity binding of the pentasaccharide of ganglioside GM1 (mentioned above as galectin-1 counterreceptor) occurred to mesothelioma [78], and GalNAc binding was different between sections of typical and atypical carcinoid tumors of the lung [79]. Using the strategic combination of carbohydrate chemistry to synthesize biologically relevant di- and oligosaccharides, e.g. the Thomsen-Friedenreich (T) antigen or histo-blood group determinants, with glycohistochemistry answered the question positively for tissue presence of respective receptors, e.g. in breast and lung cancer [80,81]. Based on analysis of 187 lung cancer cases a favorable prognostic correlation was discerned for binding of the histo-blood group A and H (but not B) trisaccharides [81]. In this respect, members of the family of galectins (galactoside-binding β-sandwich-fold proteins with a sequence signature prominently harboring a Trp moiety responsible for contact to the Gal-moiety of cognate glycans [38]) are candidate receptors, owing to their involvement in growth processes and the known reactivity to these epitopes [82,83]. Thus, results from glycocyto- and histochemistry give direction for the next step to take in analyzing the glycan-based communication, i.e. to purify the tissue lectins and work with lectin-specific antibodies (please see third section in Table 4), here illustrated exemplarily for galectins as example.

### The galectin network in tumors

Following the directives given above, sugar-specific staining guides selection of the resin-bound ligand for affinity chromatographic purification of proteins(s) with respective specificity. Glycohistochemical monitoring is thus more than a screening. The strong staining intensity specific for β-galactosides (of neoglycoproteins bearing lactose or glycoproteins after sialic acid removal from the glycan chains) in muscle biopsy specimens (itself of diagnostic value in fiber typing on fixed material [84]) prompted lectin purification using a resin presenting lactose and raising lectin-specific antibodies with the protein now termed galectin-1. The ensuing immunohistochemical work revealed the expectable similarity between the glyco- and immunohistochemical staining patterns [84]. The Ca^2+^-independent tissue reactivity to lactose moieties or the N-acetyllactosamine terminus of N-glycan chains of asialofetuin is thus attributable to galectin-1, the most prevalent member of this lectin family in muscle (mentioned above as potent growth regulator), and galectins are also found in tumors. Reflecting the apparent diversification of the ancestral gene in this lectin family [35,38], more than one galectin can be expressed in tumors, initially documented by histopathology using non-crossreactive antibodies in breast cancer [85]. Of course, the localization profiles can differ, and parameters such as degree of differentiation can affect the results to non-identical extents for the proteins tested. Consequently, galectin fingerprinting with non-crossreactive antibodies, and not a focus on a single family member, is advisable to get a clear view on effector presence.

Respective studies on colon and on head and neck cancer as well as bladder carcinoma went beyond underscoring the network concept by disclosing prognostic relevance for distinct family members, e.g. galectins-1 and -4 in colon cancer [86,87], galectin-7 in hypopharyngeal cancer [88,89] as well as galectins-2 and -8 in urothelial carcinoma [90]. Moreover, differential diagnosis and cell-lineage characterization are aided by this fingerprinting [91,92]. Combined with syntactic structure analysis, which assesses cell distribution relative to staining parameters for tumor/inflammatory cells including measuring structural entropy (for methodological details, please see [93,94]), further prognostic indicators can be delineated. Distances between tumor cells (positivity for galectin-8 at high intensity) and lymphocytes as well as between galectin-1-positive tumor cells were of prognostic relevance in testicular cancer patients with lung metastases [95]. To avoid false-negative results in such studies, special attention should be given to the possibility for *in situ* processing of a galectin and the appropriate selection of the antibody: proteolytic truncation by matrix metalloproteinases -2 and -9 of galectin-3’s N-terminal tail can remove the antigenic epitope from the lectin and hereby be the reason for making the lectin invisible.

Two lines of experimental evidence guide from this fingerprinting to the next methodological step: i) the glycophenotype analysis with plant lectins and ii) the functional correlation between receptor (lectin)/counterreceptor (glycan) expression, as outlined above, both encourage to test tissue lectins as tools. After having paved the way to these proteins in sufficient quantities by recombinant expression, galectin cyto- and histochemistry could be realized (please see fourth section in Table 4). As plant agglutinins, human lectins can readily be labeled, under activity-preserving conditions, and then employed to trace reactive sites in cells, in different types of specimens [96-101]. In tumor pathology, galectins-3 binding proved of prognostic relevance in colon and advanced laryngeal/hypopharyngeal cancer [102,103]. Interestingly, a modification of the lectin, e.g. by altering structural parameters relevant for stability or clearance [104,105], is not neutral but can change binding properties or harm activity. Combining the monitoring of distinct characteristics of the glycophenotype such as the status of α2,6-sialylation with measuring presence of galectins and their reactivity, as performed for colon cancer [28,86,87,103], along with monitoring of functionally relevant gene products such as the fibronectin receptor (α_5_β_1_-integrin), a galectin counterreceptor in carcinoma growth regulation via cell cycle arrest or anoikis induction [51,106,107], has potential to take mapping of epitope presence to function-oriented analysis. Similarly, galectin expression along with its apoptosis-inducing counterreceptor CD7 explains the clonal selection toward CD4^+^CD7^-^ T leukemic cells during progression of the Sézary syndrome [108]. The presented principles underlying the glyco- and galectin cyto- and histochemical approaches are not only instrumental for the glycobiological aspect of specimen characterization. They also have relevance beyond studying glycobiological effector pathways. Examples inspiring further applications will be given in the next section.

### Carrier-immobilized/free ligands tracing their receptors

The localization of galectins by labeled (neo)glycoconjugates documents maintained capacity for specific binding even after fixation of the specimen and its exposure to organic solvents. If this prerequisite is fulfilled for a receptor, more types of natural ligands can serve the purpose to track down presence of accessible binding sites. In the first step, a conjugation to a labeled scaffold without impairing the ligand activity is performed. Using estradiol and testosterone, attached to albumin as inert carrier, presence of steroid hormone receptors was detected in tissue sections of lung cancer and in pleural effusion of mesothelioma, here with relation to the S-phase-related tumor cell fraction [109,110]. When similarly presented by a carrier, anthracylin reactivity was revealed in sections of small cell lung cancer sections, with prognostic relevance [111]. In addition to the information obtained by the staining reaction, positivity also is an incentive for biochemical work to determine the nature of binding sites for the scaffold-presented ligands, the case study on galectin isolation from muscle mentioned above.

Mimicking the (ga)lectin histochemical approach of glycan profiling, any protein is suited for localization of binding partners, if labeling does not harm the interaction with counterreceptor(s). To achieve localization biotinylation by signal generation has been found applicable for interleukin-2 [112,113], erythropoietin [114], the epidermal growth factor [115,116], macrophage migration inhibitory factor, with prognostic relevance in carcinoid tumors of the lung [79], and sarcolectin interacting with this broad-spectrum pro-inflammatory cytokine [81,117,118]. In order to select the least activity-reducing type of modification for labeling, systematic study of the influence of amino acid modification with group-specific reagents on the interaction is advised. In principle, this approach extends the scope of immunohistochemical localization, especially in cases when a ligand can bind to several distinct receptors or when characterizing the range of interaction partners has not yet been taken to the biochemical level.

## Conclusions

Carbohydrates can form oligo- and polymers, with equally wide distribution as that of nucleic acids and proteins. Biochemically, they are more than fuel or concrete for cell walls. The consideration of the chemical properties of sugars explains their amazing capacity to turn the ‘letters’ of this alphabet (monosaccharides) into an unsurpassably large array of ‘words’. While being central to the enormous versatility of sugar-based coding, this structural complexity poses an extraordinary challenge for compound analysis, much greater than for any other class of biopolymer. Fittingly, respective methods have become available only recently. Physiologically, the molecular meaning of cellular glycan determinants can be translated into responses by receptors (lectins). Evidently, the glycophenotype of cells has a functional dimension, nourishing the expectations for biomedical relevance of its characterization.

Based on the concept of the sugar code outlined above four technical approaches enable histopathologists to define respective aspects cyto-and histochemically:

1. Glycan profiling by carbohydrate-specific antibodies or plant/invertebrate lectins

2. Profiling of carbohydrate-binding sites by (neo)glycoconjugates

3. Immunohistochemical detection of lectins and fingerprinting for family members in an emerging network

4. Profiling cognate sites by endogenous lectins (in combination with 3. enabling colocalization)

Taking this step-by-step approach is currently revealing correlations between clinical and glycopathological parameters, encouraging further efforts. Work *in vitro* substantiates the assumed effector functionality of lectins by targeting distinct counterreceptors such as an integrin, an adhesion molecule or a ganglioside. These findings in turn inspire further investigations on clinical specimens. For monitoring serum samples, the lectin-reactive clycans of the cellular clycoconjugate(s) then can become functional glycobiomarkers. Of note, the approach with carrier-immobilized ligands and labeled receptor proteins (here lectins) can find application for epitope-specific localization studies beyond glycopathology.

## Competing interests

The authors declare that they have no competing interests.

## Authors’ contribution

HJG and KK shared work on design and preparation of the review. Both authors read and approved the final manuscript.
